# Reduced mitochondrial D-loop methylation levels in sporadic amyotrophic lateral sclerosis

**DOI:** 10.1186/s13148-020-00933-2

**Published:** 2020-09-11

**Authors:** Andrea Stoccoro, Adam R. Smith, Lorena Mosca, Alessandro Marocchi, Francesca Gerardi, Christian Lunetta, Cristina Cereda, Stella Gagliardi, Katie Lunnon, Lucia Migliore, Fabio Coppedè

**Affiliations:** 1 Department of Translational Research and of New Surgical and Medical Technologies, Lab. of Medical Genetics, University of Pisa, Medical School, Via Roma 55, 56126 Pisa, Italy; 2 University of Exeter Medical School, College of Medicine and Health, Exeter University, Exeter, UK; 3 Medical Genetics Unit, Department of Laboratory Medicine, ASST Grande Ospedale Metropolitano Niguarda, Milan, Italy; 4 NEMO Clinical Center, Fondazione Serena Onlus, Milan, Italy; 5 Genomic and Post-Genomic Center, IRCCS Mondino Foundation, Via Mondino 2, 27100 Pavia, Italy

**Keywords:** Amyotrophic lateral sclerosis, Epigenetics, Mitoepigenetics, D-loop mitochondrial region, Mitochondrial DNA methylation, Mitochondrial DNA copy number, Sporadic ALS, *SOD1*, *C9orf72*

## Abstract

**Background:**

Mitochondrial dysregulation and aberrant epigenetic mechanisms have been frequently reported in neurodegenerative diseases, including amyotrophic lateral sclerosis (ALS), and several researchers suggested that epigenetic dysregulation in mitochondrial DNA (mtDNA) could contribute to the neurodegenerative process. We recently screened families with mutations in the major ALS causative genes, namely *C9orf72*, *SOD1*, *FUS*, and *TARDBP*, observing reduced methylation levels of the mtDNA regulatory region (D-loop) only in peripheral lymphocytes of *SOD1* carriers. However, until now no studies investigated the potential role of mtDNA methylation impairment in the sporadic form of ALS, which accounts for the majority of disease cases. The aim of the current study was to investigate the D-loop methylation levels and the mtDNA copy number in sporadic ALS patients and compare them to those observed in healthy controls and in familial ALS patients. Pyrosequencing analysis of D-loop methylation levels and quantitative analysis of mtDNA copy number were performed in peripheral white blood cells from 36 sporadic ALS patients, 51 age- and sex-matched controls, and 27 familial ALS patients with germinal mutations in *SOD1* or *C9orf72* that represent the major familial ALS forms.

**Results:**

In the total sample, D-loop methylation levels were significantly lower in ALS patients compared to controls, and a significant inverse correlation between D-loop methylation levels and the mtDNA copy number was observed. Stratification of ALS patients into different subtypes revealed that both *SOD1*-mutant and sporadic ALS patients showed lower D-loop methylation levels compared to controls, while *C9orf72*-ALS patients showed similar D-loop methylation levels than controls. In healthy controls, but not in ALS patients, D-loop methylation levels decreased with increasing age at sampling and were higher in males compared to females.

**Conclusions:**

Present data reveal altered D-loop methylation levels in sporadic ALS and confirm previous evidence of an inverse correlation between D-loop methylation levels and the mtDNA copy number, as well as differences among the major familial ALS subtypes. Overall, present results suggest that D-loop methylation and mitochondrial replication are strictly related to each other and could represent compensatory mechanisms to counteract mitochondrial impairment in sporadic and *SOD1*-related ALS forms.

## Background

Amyotrophic lateral sclerosis (ALS) is the most common type of motor neuron disease, characterized by the progressive degeneration of both upper and lower motor neurons, which leads to muscle atrophy, gradual paralysis, and finally death, usually as a result of respiratory failure 5 years after disease onset [1]. The pathological processes that lead to neuronal death are not yet completely understood, but numerous mechanisms, including oxidative stress, mitochondrial dysfunction, impaired axonal transport, protein and RNA aggregation, excitotoxicity, and neuroinflammation, have been linked to ALS [2]. The only ALS drugs available are riluzole, which slows the progression of symptoms and increases life expectancy by only about 3 months, and edaravone, a potent antioxidant compound that has only recently been approved by the United States Food and Drug Administration (USFDA) for use in ALS patients that slightly reduces the disease progression in a subgroup of patients with specific features [3–5].

Approximately 10% of ALS cases follow a Mendelian, mostly autosomal dominant, inheritance pattern (familial ALS), with at least two diagnoses in the family lineage [6]. To date, more than 20 genes have been linked to familial ALS, including *C9orf72*, superoxide dismutase 1 (*SOD1*), TAR DNA-binding protein (*TARDBP*), and fused in sarcoma (*FUS*) that account for the majority of the cases, and representing 40%, 20%, 1–5%, and 1–5% of cases, respectively. However, the majority of ALS (more than 90%) is sporadic (sporadic ALS), of whom 10–15% carrying one or two of the previously described gene alterations, likely resulting from complex gene-environment interactions not yet completely clarified [2]. Dozens of genes and many non-genetic factors, including advanced age, being male, infections, intense physical activity, body mass index, nutritional state, and exposure to environmental factors, such as organophosphates and heavy metals, have been linked to sporadic ALS risk [7, 8]. Epigenetic mechanisms, dysregulated either by environmental factors or by the presence of mutations in ALS-causative genes, are increasingly believed to contribute to ALS pathogenesis and progression [9]. Particularly, several studies revealed global and gene-specific epigenetic changes in ALS animal models and in tissues derived from both familial and sporadic ALS patients [10–15]. Most of these studies were focused on nuclear genes and on the epigenetic landscape of the nuclear genome, but recent evidence suggests that epigenetic modifications to the mitochondrial genome could also contribute to neurodegeneration [16].

In this regard, recent studies have highlighted the potential role of dysregulation in mitochondrial DNA (mtDNA) methylation and hydroxymethylation levels in the etiology of several human diseases, including cardiovascular diseases, cancer, and neurodegenerative diseases [16–18]. The majority of these studies investigated DNA methylation levels of the mtDNA regulatory region (D-loop), which plays a fundamental role in regulating mtDNA replication and transcription, revealing that D-loop methylation levels were dynamically dysregulated with disease progression in animal models of Alzheimer’s disease (AD) and were significantly different from those seen in healthy controls in human post-mortem AD brains and in peripheral blood cells of living AD patients [19, 20], as well as in transgenic AD mice [21]. A significant reduction of D-loop methylation levels was also observed post-mortem in the *substantia nigra* of individuals with Parkinson’s disease compared to healthy controls [19]. Regarding ALS, reduced D-loop methylation levels were detected in spinal cord and skeletal muscle cells of human-*SOD1* transgenic ALS mice [22]. Furthermore, we recently investigated ALS families with *SOD1*, *C9orf72*, *FUS*, or *TARDBP* pathogenetic variants, observing that the methylation levels of the mitochondrial D-loop region were significantly decreased only in peripheral blood lymphocytes of carriers of ALS-linked *SOD1* variants, either ALS patients or pre-symptomatic carriers. Moreover, D-loop methylation levels resulted inversely correlated with the mtDNA copy number, suggesting that among familial ALS cases, changes in mitochondrial epigenetics leading to increased mtDNA replication could be particularly relevant for those with an impaired antioxidant defense, due to the presence of *SOD1* mutations [23]. However, nothing is still known concerning the mitochondrial epigenetics of sporadic ALS cases, which represent the vast majority of ALS patients.

To further address the potential involvement of mtDNA methylation in ALS pathogenesis, in the current study, we quantified the DNA methylation levels at the D-loop regulatory region and the mtDNA copy number in peripheral white blood cells from sporadic ALS patients and compared their levels with healthy controls and familial ALS patients.

## Results

In the present study, a total of 114 individuals, including 63 ALS patients and 51 age- and sex-matched healthy controls, were enrolled (Table 1). The ALS group comprises 14 patients with pathogenic variants in the *SOD1* gene, 13 patients with a pathogenic *C9orf72* repeat expansion, and 36 sporadic cases negative for the presence of pathogenic alterations in the major ALS genes, namely *SOD1*, *C9orf72*, *FUS*, and *TARDBP*. Bisulfite pyrosequencing was used to quantify DNA methylation in the mitochondrial D-loop region, and quantitative PCR (qPCR) was used to evaluate the mtDNA copy number.

**Table 1:** Demographic characteristics of the study population

Main groups	ALS subtypes	Age at sampling	Sex (F/M)
Control subjects (*n* = 51)		67.0 ± 13.5	24/27
ALS patients (*n* = 63)	Sporadic ALS (*n* = 36) *SOD1*-ALS (*n* = 14) *C9orf72*-ALS (*n* = 13)	64.2 ± 11.1	30/33
*p* value		0.22^a^	0.95^b^

^a^Student’s *t* test

^b^Fisher exact test

### Age at sampling and sex effect on D-loop methylation levels and on mtDNA copy number

**Fig. 1:**
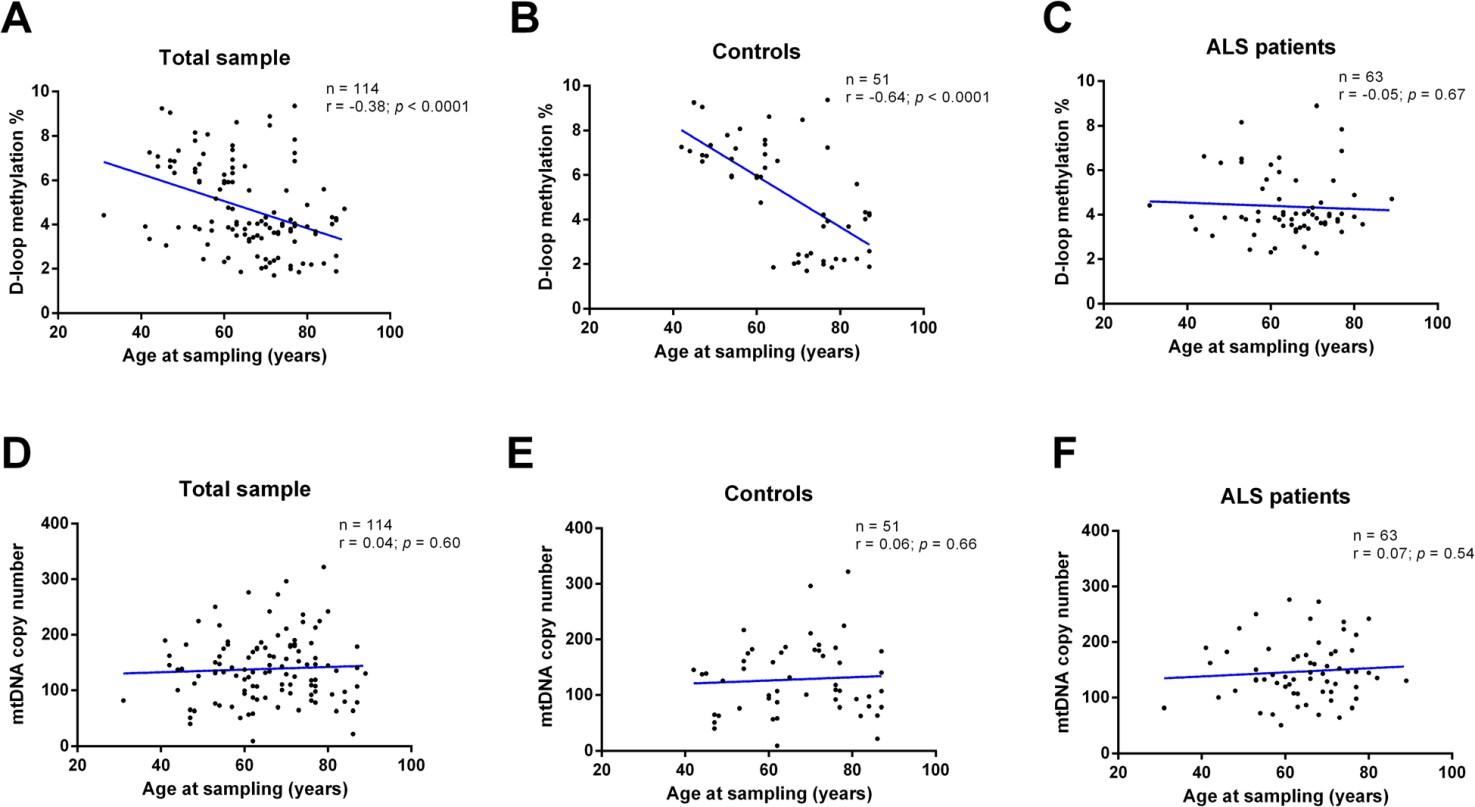
Correlation between age at sampling and D-loop methylation levels in total sample (*n* = 114, **a**), in control subjects (*n* = 51, **b**) and in ALS patients (*n* = 63, **c**). Correlation between age at sampling and mtDNA copy number in total sample (*n* = 114, **d**), in control subjects (*n* = 51, **e**), and in ALS patients (*n* = 63, **f**). The correlation between age at sampling and D-loop methylation levels was analyzed using Pearson’s correlation coefficient

Figure 1 shows the effect of age at sampling on D-loop methylation levels and on the mtDNA copy number. An inverse correlation between age at sampling and D-loop methylation levels (*r* = − 0.38; *p* < 0.0001, 95% CI − 0.52 to − 0.21) was observed in the total sample (Fig. 1a). This correlation was driven by control subjects (*r* = − 0.64; *p* < 0.0001, 95% CI − 0.78 to − 0.44; Fig. 1b), while no significant correlation between D-loop methylation levels and age at sampling was observed in ALS patients (*r* = − 0.05; *p* = 0.67, 95% CI − 0.29 to 0.19; Fig. 1c). No significant correlation between age at sampling and mtDNA copy number was detected (*r* = 0.04; *p* = 0.60, 95% CI − 0.13 to 0.23) in the total sample (Fig. 1d), as well as in control subjects (*r* = 0.04; *p* = 0.60, 95% CI − 0.21 to 0.33; Fig. 1e) and in ALS patients (*r* = 0.07; *p* = 0.54, 95% CI − 0.17 to 0.31; Fig. 1f).

**Fig. 2:**
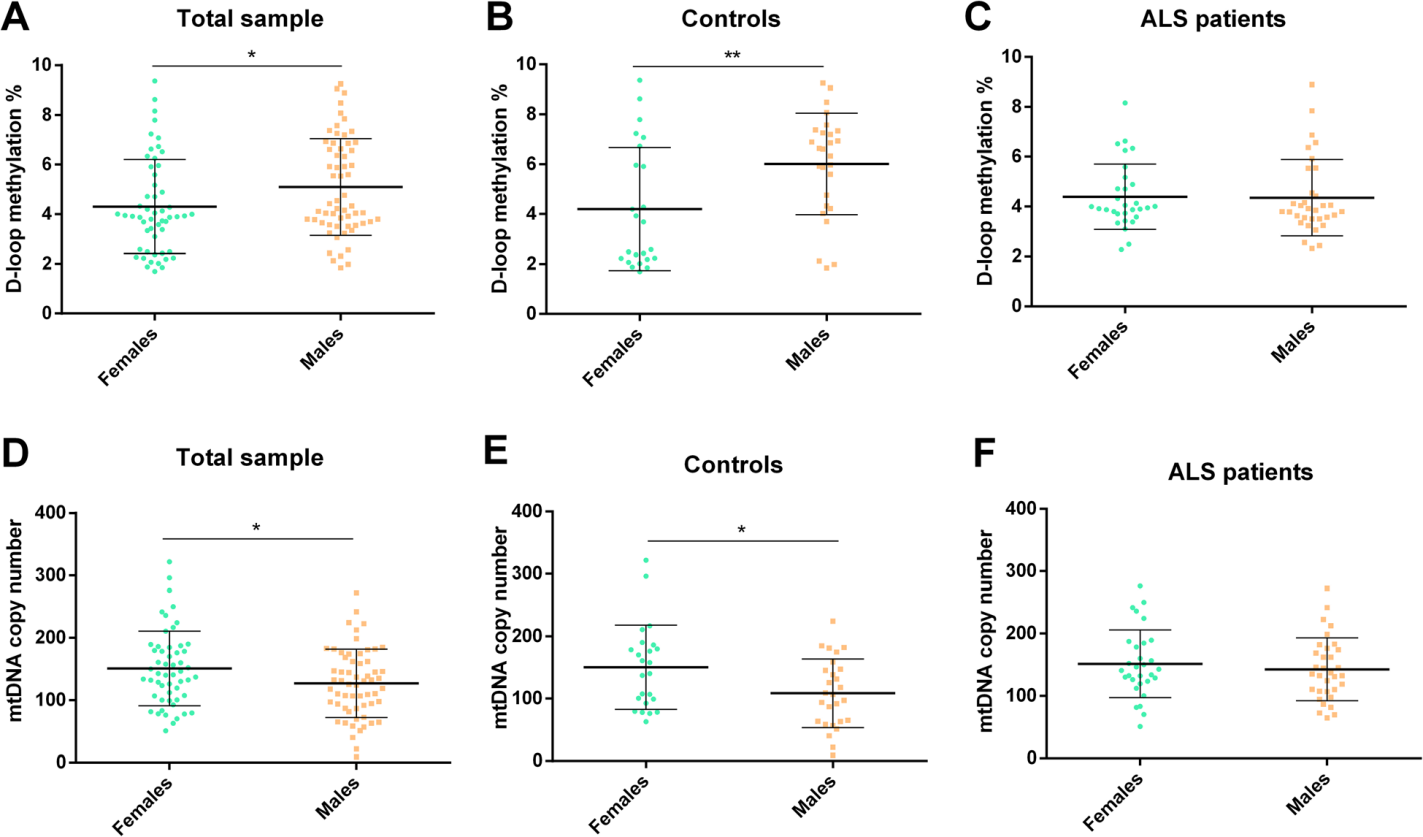
Effect of sex on D-loop methylation levels in total sample (*n* = 114, **a**), in control subjects (*n* = 51, **b**), and in ALS patients (*n* = 63, **c**). Effect of sex on mtDNA copy number in total sample (*n* = 114, **d**), in control subjects (*n* = 51, **e**), and in ALS patients (*n* = 63, **f**). Statistical analysis was performed by means of ANCOVA, using age at sampling as covariate. Data are expressed as means ± standard deviation (SD). *p < 0.05; **p < 0.01

Figure 2 shows the effect of sex on D-loop methylation levels and on the mtDNA copy number. In the total sample (Fig. 2a), the D-loop methylation levels were higher in males (5.09 ± 1.85%) compared to those in females (4.30 ± 1.91%) in a statistically significant manner (*p* = 0.03, 95% CI − 1.50 to − 0.07). However, sample stratification into ALS patients and controls revealed that the sex effect was only seen in controls. Indeed, in control subjects (Fig. 2b), the D-loop methylation levels were higher in males compared to those in females (6.01 ± 2.23% vs. 4.20 ± 2.20%; *p* = 0.006, 95% CI − 3.07 to − 0.53), while in ALS patients (Fig. 2c), there was no statistical difference between males and females (4.35 ± 1.37% vs. 4.39 ± 1.42%; *p* = 0.91, 95% CI − 0.68 to − 0.76). Figure 2d shows that in the total sample, the mtDNA copy number was significantly higher in females compared to that in males (150.84 ± 57.39 vs. 127.22 ± 57.38; *p* = 0.03, 95% CI 2.27 to 44.96), but again the sex difference was driven by control subjects. Indeed, higher mtDNA copy number in females (150.10 ± 61.38) compared to that in males (108.36 ± 61.36) was observed in control subjects (*p* = 0.02, 95% CI 7.12 to 76.34; Fig. 2e), but not in ALS patients (Fig. 2f) in which, although the mtDNA copy number was slightly higher in females (151.44 ± 52.36) than in males (142.65 ± 52.33), the difference was not statistically significant (*p* = 0.50, 95% CI − 17.63 to 35.21).

### Correlation between D-loop methylation levels and the mtDNA copy number

**Fig. 3:**
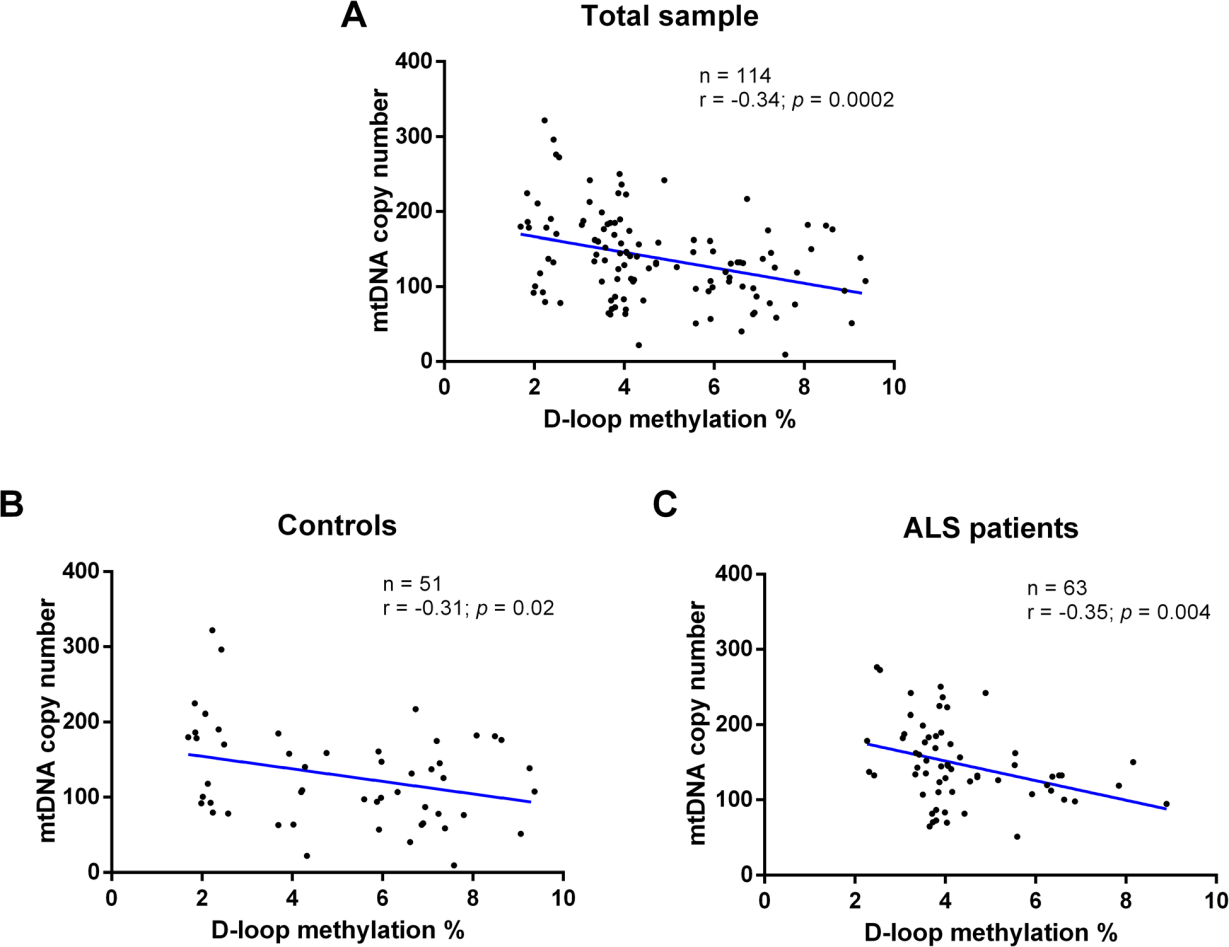
Correlation between mtDNA copy number and D-loop region methylation levels in total sample (*n* = 114, **a**), in control subjects (*n* = 51, **b**), and in ALS patients (*n* = 63, **c**). The correlation between mtDNA copy number and D-loop methylation levels was analyzed using Pearson’s correlation coefficient.

A significant inverse correlation between D-loop methylation levels and mtDNA copy number was observed in the total sample (*r* = − 0.34; *p* = 0.0002, 95% CI − 0.49 to − 0.17) (Fig. 3a). This inverse correlation remained significant also in control subjects (*r* = − 0.31; *p* = 0.02, 95% CI − 0.54 to − 0.04) (Fig. 3b) and in the ALS patients (*r* = − 0.35; *p* = 0.004, 95% CI − 0.55 to − 0.11) (Fig. 3c).

### D-loop methylation levels and mtDNA copy number in ALS patients and controls

**Fig. 4:**
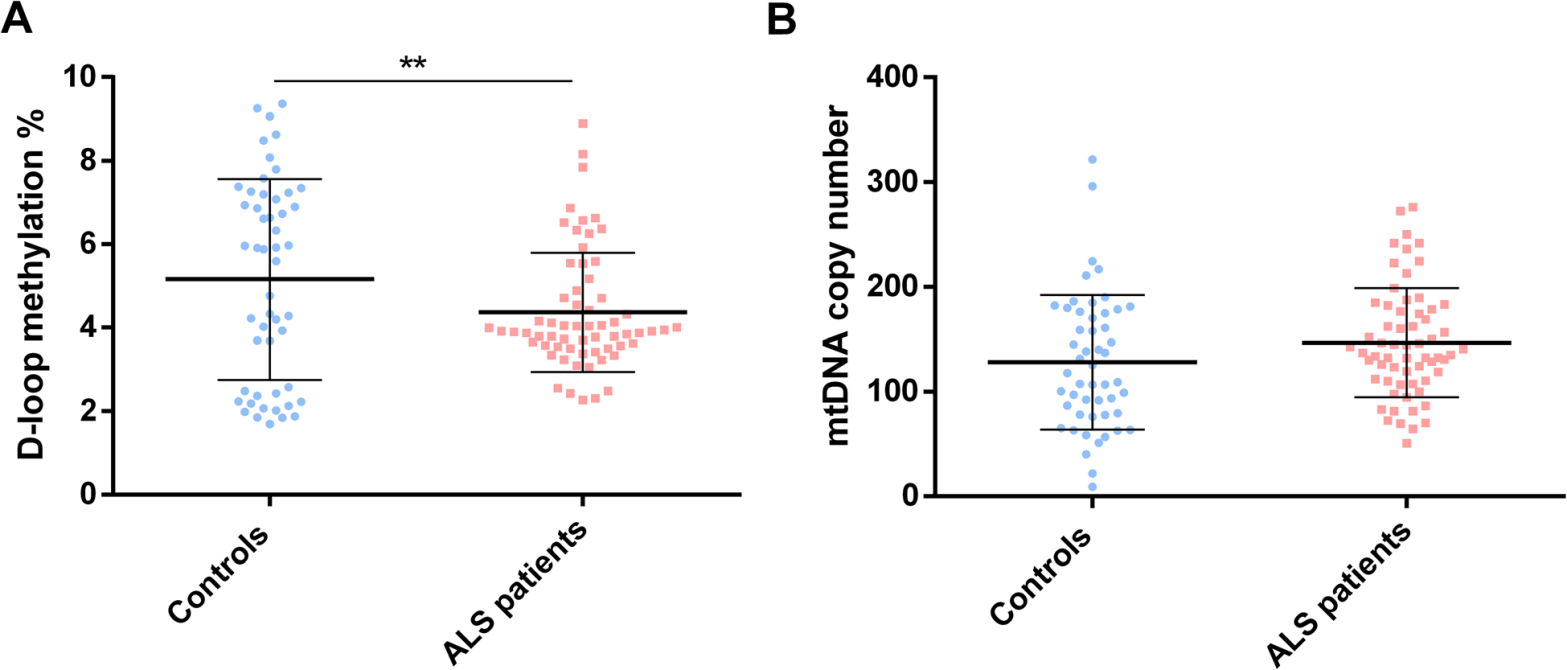
D-loop region methylation levels (**a**) and mtDNA copy number (**b**) in controls (*n* = 51) and ALS patients (*n* = 63). Data are expressed as means ± SD. Statistical analysis was performed by means of ANCOVA including age at sampling and sex as covariates. **p < 0.01

Overall, we observed that D-loop methylation levels were significantly higher in control subjects than in ALS patients (5.25 ± 1.71% vs. 4.29 ± 1.66%, *p* = 0.004, 95% CI 0.30 to 1.61) (Fig. 4a). The mtDNA copy number showed a trend to be higher in ALS patients compared to control subjects, although this was not statistically significant (147.04 ± 57.22 vs. 127.75 ± 57.27, *p* = 0.07, 95% CI − 40.75 to 2.16) (Fig. 4b).

**Fig. 5:**
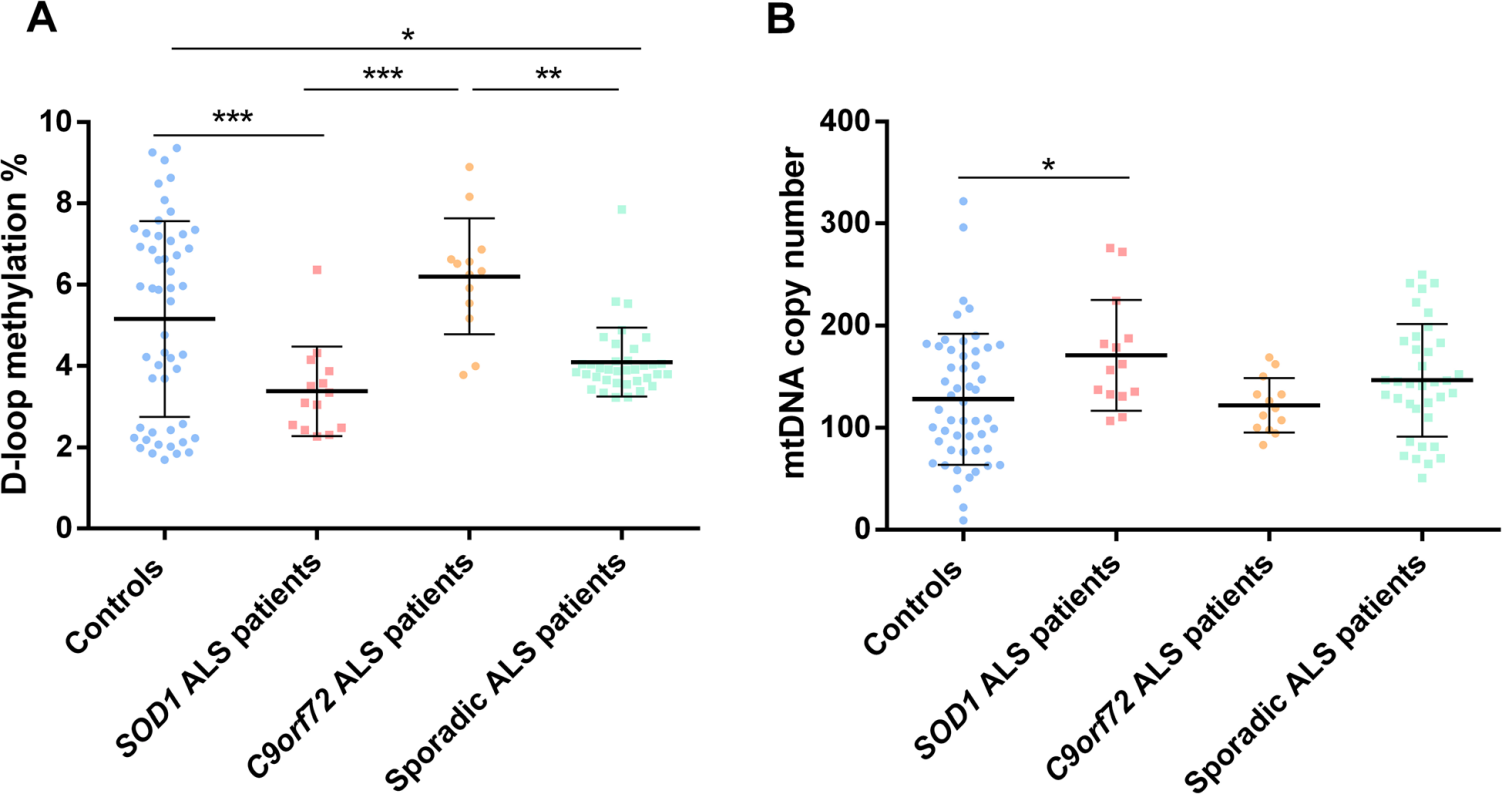
D-loop region methylation (**a**) and mtDNA copy number (**b**) in controls (*n* = 51), *SOD1*-ALS (*n* = 14), *C9orf72*-ALS (*n* = 13), and sporadic ALS patients (*n* = 36). Data are expressed as means ± SD. Statistical analysis was performed by means of ANCOVA, using age at sampling and sex as covariates. Only *p* values that survived correction for multiple comparisons are shown. *p < 0.05; **p < 0.01; ***p < 0.001

We next investigated if D-loop methylation levels and mtDNA copy number varied in different ALS subtypes (Fig. 5). We observed that both *SOD1* (2.98 ± 1.60%) and sporadic ALS patients (4.22 ± 1.56%) had lower D-loop methylation levels compared to both control subjects (5.25 ± 1.57%; *p* = 0.0001, 95% CI 0.94 to 3.57 and *p* = 0.02, 95% CI 0.09 to 1.95, respectively) and *C9orf72* ALS patients (5.91 ± 1.58%; *p* < 0.0001, 95% CI − 4.58 to − 1.27 and *p* = 0.01, 95% CI 0.28 to 3.10, respectively). No significant difference was observed between healthy controls and *C9orf72* ALS patients (*p* = 1.00, 95% CI − 2.02 to 0.68) (Fig. 5a). The mtDNA copy number showed the opposite direction of effect and was significantly higher in *SOD1* ALS patients (175.02 ± 56.83) with respect to control subjects (127.75 ± 56.27; *p* = 0.04, 95% CI − 93.49 to − 1.04), while it showed a non-significant increase in sporadic ALS patients (145.33 ± 56.28; *p* = 0.91, − 50.41 to 15.23) compared to controls, and was similar between controls and *C9orf72* ALS patients (121.66 ± 56.85; *p* = 1.00, 95% CI − 41.58 to 53.75) (Fig. 5b).

## Discussion

In the present study, we investigated D-loop methylation levels and mtDNA copy number in peripheral white blood cells from a group of 63 ALS patients, including 36 sporadic patients negative for the major ALS gene alterations and 27 familial cases with a germinal mutation in *SOD1* or *C9orf72* genes, and 51 healthy sex- and age-matched controls. In the total sample, D-loop methylation levels were significantly lower in ALS patients compared to control subjects, and the mtDNA copy number showed a non-statistically significant tendency to be higher in the first group. Indeed, a significant inverse correlation between D-loop methylation levels and the mtDNA copy number was observed. We also observed that D-loop methylation levels decreased with increasing age at sampling and were higher in males compared to females, while the mtDNA copy number was higher in females than in males; however, after stratification of the sample into ALS patients and controls, age and sex effects were evident in control subjects, but not in ALS patients. Stratification of the ALS samples into sporadic and familial cases revealed that both *SOD1*-mutant and sporadic ALS patients showed lower D-loop methylation levels compared to control subjects. By contrast, ALS *C9orf72*-expansion carriers showed similar D-loop methylation levels to healthy controls, and significantly higher than those observed in sporadic or *SOD1*-mutant ALS patients. The copy number of the mtDNA showed an opposite direction of effect to D-loop methylation levels, being significantly higher in *SOD1*-mutant ALS patients than in controls.

Accumulating evidence suggests impaired mtDNA methylation in patients with neurodegenerative diseases, and particularly in the regulatory D-loop region [19–23]. Indeed, altered D-loop methylation levels have been observed in cortical regions of AD patients and transgenic AD mice [19, 21], in the *substantia nigra* of PD patients [19], and in the peripheral blood cells of sporadic AD patients [20]. Concerning ALS, altered methylation levels in the mitochondrial 16S rRNA gene and in the D-loop region were detected in the spinal cord and skeletal muscle of human-*SOD1* transgenic ALS mice, and these changes were paralleled by a significant reduction of the mitochondrial DNA methyltransferase 3a (Dnmt3a) protein levels [22]. More recently, we investigated D-loop methylation levels in several ALS families with mutations in *SOD1*, *C9orf72*, *FUS*, or *TARDBP* genes and compared their levels among affected patients, pre-symptomatic carriers, and non-carrier family members, observing that the methylation levels of the mitochondrial D-loop region were significantly decreased only in peripheral blood DNA of carriers of ALS-linked *SOD1* mutations, either ALS patients or pre-symptomatic carriers, with respect to non-carriers of ALS-linked mutations; furthermore, an inverse correlation between D-loop methylation levels and the mtDNA copy number was observed [23]. The present study compared sporadic ALS patients, familial ALS patients, and unrelated healthy controls. We confirmed decreased D-loop methylation levels in *SOD1-*mutant ALS patients and revealed, for the first time, decreased mtDNA methylation in peripheral blood cells of sporadic ALS patients compared to age- and sex-matched control subjects. On the other hand, ALS *C9orf72*-expansion carriers showed similar D-loop methylation levels to healthy controls and significantly higher than those observed in sporadic or *SOD1*-mutant ALS patients. Some investigations demonstrated that different ALS-linked genes have diverse effects on mitochondrial dynamics and function [24, 25], and previous studies suggested that nuclear DNA methylation signatures too can differ among patients with a different ALS genetic etiology. Particularly, Ebbert and coworkers observed differential DNA methylation patterns in both the cerebellum and the frontal cortex of *C9orf72*-associated and sporadic ALS patients compared to those of control subjects [26]. In that study, it was also observed that, although some differentially methylated regions were shared between the two ALS patient sub-groups, the majority of the differentially methylated regions were unique to *C9orf72*-associated or sporadic ALS [26]. More recently, Tarr and coworkers (2019) analyzed the blood methylome and transcriptome of a longitudinal ALS-discordant cohort comprising monozygotic triplets and twins and representing the three most common types of ALS, namely *C9orf72*-linked ALS, *SOD1*-linked ALS, and sporadic ALS. Co-twin analyses revealed that older twins showed a consistent difference in DNA methylation patterns between the affected and the unaffected twins [14]. Moreover, by analyzing DNA methylation in the genes commonly altered, it was evident that sporadic ALS patients were more similar to *SOD1*-related cases (12 altered genes in common), than sporadic ALS with *C9orf72*-related (1 gene in common), or *SOD1* and *C9orf72* (0 genes in common) cases. Results from the present study suggest that the mtDNA D-loop methylation levels in sporadic ALS patients are more similar to *SOD1*-mutant patients than to *C9orf72*-ALS patients, given that both sporadic and *SOD1*-related ALS patients had lower mitochondrial methylation levels compared to control subjects and both differed significantly from the methylation levels observed in *C9orf72*-ALS patients. Present data also confirm our previous investigation in familial ALS, revealing that among carriers of the major ALS-causative genes, namely *SOD1*, *C9orf72*, *FUS*, and *TARDBP*, D-loop methylation levels were significantly reduced only in *SOD1* carriers [23]. Collectively, these studies suggest that each ALS-causative gene contributes to nuclear and mitochondrial DNA methylation alteration in a different manner [14, 23, 26]. Furthermore, it has been reported that ALS-causative genes are able to interact with epigenetic writer and reader proteins [12]. Indeed, in vitro and in vivo studies reported that mutant *SOD1*, as well as *FUS* and *C9orf72* genes, encode for proteins that are able to interact in different ways with histone acetyltransferases (HATs) and methyltransferases (HMTs), thus inducing global changes to the histone code [27–30], while TDP43, encoded by *TARDBP* gene, is involved in RNA-related metabolism, and it is directly implicated in miRNA biogenesis and in the regulation of long non-coding RNAs [31]. So, it is plausible that different ALS-causative genes induce specific epigenetic alterations in both the nuclear and mitochondrial genomes.

In the current study, we detected an inverse correlation between D-loop methylation levels and the mtDNA copy number, an observation that has been repeatedly reported in the literature [16]. Indeed, a significant inverse correlation between D-loop methylation levels and the copy number of the mtDNA has been observed in human and mouse cell cultures [32, 33], in human peripheral blood cells [23, 34–37], in colorectal cancer tissues [38], and in the human placenta [39]. Data obtained in the current study are in agreement with those studies, further suggesting that variations in D-loop methylation levels could modulate the mtDNA copy number. Indeed, the D-loop is a non-coding mitochondrial region of about 1.1 kb, critical for both mitochondrial replication and transcription [40], which contains the promoter regions of the mitochondrial genes and the origin of replication of the heavy mtDNA strand [41]. Moreover, it is widely accepted that D-loop facilitates mtDNA replication by maintaining an open structure of mtDNA and this makes the D-loop a likely candidate for epigenetic modifications, which can have a major impact on the expression and replication of mitochondria [32, 41]. In this way, it has been reported that DNA methyltransferase 1 (DNMT1), the enzyme responsible for maintenance of DNA methylation, is able to bind to the mitochondrial D-loop region inducing repression of transcription of mtDNA genes and reducing mtDNA copy number [42–44]. Further evidence of a potential contribution of D-loop methylation levels in regulating mtDNA copy number and gene expression comes from studies in colorectal cancer cell lines showing that demethylation of the D-loop region, induced by the demethylating agent 5-aza-2′-deoxycytidine (5-Aza), was associated with an elevated mtDNA copy number [38, 45]. So it is likely that D-loop methylation could regulate its own activity, similarly to what occurs in the promoter of nuclear genes. Given that the mitochondria play fundamental roles in numerous cellular functions, and that their alterations are involved in several pathologies, numerous authors searched for altered mtDNA methylation in human diseases, and increasing evidence suggests that an increase in mtDNA copy number and mitochondrial transcription, resulting from mtDNA methylation changes, might be a compensatory mechanism to counteract oxidative stress and/or an increased ATP demand under conditions of oxidative stress and metabolic dysfunctions such as those resulting from placental insufficiency [46], cancer [38], obesity, and diabetes [37]. Indeed, we previously suggested that the reduced D-loop methylation levels leading to an increased mtDNA copy number in carriers of ALS-linked *SOD1* mutations could represent a compensatory mechanism to counteract the increased oxidative stress, impaired mitochondrial respiration, and increased mtDNA damage in those subjects [23]. In this regard, it has also been recently demonstrated that DNA repair genes are hypomethylated and overexpressed to counteract oxidative DNA damage in human ALS motor neurons with *SOD1* mutations [47]. Defective mitochondrial respiration and ATP production are also well documented in the spinal cord and lymphocytes of sporadic ALS patients, so that the reduced D-loop methylation levels that we observed in the present study could represent a compensatory mechanism to counteract the mitochondrial impairment in sporadic ALS too. So, the results of the present study further suggest that the methylation levels of mitochondrial D-loop region could regulate mtDNA replication due to mitochondrial impairment.

The analysis of mtDNA content revealed that the mtDNA copy number showed a tendency to be higher in peripheral blood of ALS patients compared to controls, although this difference was statistically significant only in *SOD1*-mutant carriers. Similarly, a previous study by us in ALS families revealed increased mtDNA content in the peripheral blood of the patients respect to their unaffected relatives [23]. Also previous evidence by other authors showed mitochondrial impairment, including mitochondrial depolarization, altered mtDNA gene expression, and increase in mtDNA platelet content, in peripheral blood of ALS patients [48–50]. It has been suggested that the increase in mtDNA content in ALS tissues could be a consequence of several mitochondrial alterations, including a compensatory phenomenon for altered activity of the electron transport chain, increase in oxidative stress, maladaptive of an insufficient homeostatic response to restore normal mitochondrial function, and altered mitochondrial fission and fusion [24, 50, 51]. Further studies should be performed to better understand the clinical significance of the mtDNA copy number evaluation in peripheral blood of ALS patients. We must also acknowledge that the relatively small sample size of ALS patients and controls used in the present study reduces the power to detect small mtDNA copy number differences among groups, so that future studies empowered in sample size are required to better address this issue.

In the current study, we also observed significant effects of age and sex on D-loop methylation levels. However, after stratification of the samples into ALS patients and controls, the contributions of age and sex on D-loop methylation levels were highly significant in control subjects, while they were not evident in ALS patients. This observation suggests that the mtDNA methylation and copy number impairment observed in ALS patients are strictly linked to the disease, and are probably driven by the necessity to counteract ALS-related mitochondrial impairments [1]. Carefully looking at Fig. 5 reveals that the distribution of D-loop methylation data in ALS patients follows clusters according to the ALS subtype, lower levels in *SOD1-*mutants, intermediate levels in sporadic ALS, and higher levels in *C9orf72* mutants, suggesting that the disease subtype is the major determinant of those levels. By contrast, D-loop methylation levels in controls have a more widespread distribution and respond to age and sex effects. Several authors have reported a correlation between mtDNA methylation and age in healthy individuals, for example, Dzitoyeva et al. [52] observed that 5-hmC levels in the mtDNA decreased in the frontal cortex of mice during aging, and this event was associated with increased expression of various mtDNA genes. In a study performed in peripheral blood from 381 individuals across a range of ages (38–107 years), the methylation levels of the *MT-RNR1* gene were positively associated with increasing age, and a 9-year-long follow-up survey showed that subjects with high methylation levels exhibit a mortality risk significantly higher than subjects with low *MT-RNR1* methylation levels [53]. Another study performed in peripheral blood of 82 individuals aged 18–91 years identified two CpG sites located within the *MT-RNR1* gene that are inversely correlated with subjects age [54]. Regarding gender, present results show that healthy females have lower mtDNA methylation levels compared to males. Interestingly, an inverse trend was observed for the mtDNA copy number analysis, which showed that healthy females have higher mtDNA copy number compared to males. These sex effects were not seen in ALS patients, suggesting the existence of compensatory mechanisms in male ALS patients to foster an increase in mtDNA copy number. The effect of sex on nuclear DNA methylation is well known, and it has been suggested that this difference could be largely due to hormonal factors [55]. Little is still known concerning sex differences in mtDNA methylation levels, even if D’Aquila and coworkers observed that although there were no differences in *MT-RNR1* gene methylation levels between females and males, the detected age-related increase of *MT-RNR1* methylation levels was more evident in women compared to men [53]. However, the estrogenic control of mitochondrial metabolism is well documented, and it is believed to be partially involved in sex-based differences in human diseases [56]. Indeed, in agreement with present data, previous studies in human blood cells have shown that females have higher mtDNA copy number than males, and this is likely resulting from hormonal differences fostering the antioxidant defense [57, 58].

Although DNA methylation impairment has been frequently reported in neurodegenerative diseases, including AD, PD, and ALS, the causality role of aberrant DNA methylation in the onset of these diseases remains unclear. In fact, it is still not established if the DNA methylation impairment is a cause or a consequence of the neurodegenerative process. However, it has been reported that early-life exposure to environmental risk factors are able to induce epimutations that lead to neurodegeneration in later life [59, 60], thus suggesting the causative role of epimutations in disease onset. Moreover, it is well known that mitochondria impairment is an early event of neurodegeneration. For example, gene expression analyses revealed that even in the early stages of AD, prior to the clinical diagnosis of the disease, many of the nuclear genes encoding subunits involved in oxidative phosphorylation (OXPHOS) are downregulated particularly in the brain regions most vulnerable to AD pathology [61, 62], and it has been observed a significant reduction in OXPHOS gene expression in white blood cells in subjects with mild cognitive impairment (MCI), some of which were subsequently found to have prodromal AD [63, 64]. Regarding ALS, mitochondrial alteration was reported to occur in early disease stages in in vivo models indicating that this is an upstream source of degeneration rather than a consequence of the disease [65, 66]. In our previous study, we observed a significant decrease in D-loop region methylation levels in both presymptomatic and symptomatic *SOD1* mutant carriers, suggesting that D-loop methylation alteration is an early event at least in individuals with ALS-linked *SOD1* mutations and precedes the onset of the disease symptoms and likely acting as a compensatory mechanism to counteract SOD1 deficiency in those individuals [23]. Studies in transgenic AD mice have also shown that D-loop methylation impairments are already detectable in the early stages of the disease in animal brains, but are also dynamically regulated as the disease progresses [19]. Many investigators have also shown that D-loop demethylation can result from impaired DNMT expression or activity, and is paralleled by an increased expression of mtDNA genes [22, 33, 35, 38]. Unfortunately, we do not have RNA samples from our patients to further address these issues. Longitudinal studies and cell culture studies are therefore required to investigate mtDNA methylation and copy number in ALS patients, their causative mechanisms, their functional consequences on gene expression and mitochondrial dynamics, and their potential clinical utility.

## Conclusions

In conclusion, the present study reveals a lower D-loop methylation level in sporadic ALS patients compared to healthy matched controls and confirms previous evidence of an inverse correlation between D-loop methylation levels and the mtDNA copy number as well as differences among major ALS subtypes. Further studies are required to clarify the functional consequences of these findings.

## Methods

### Study population

In the current study, blood samples were collected from 114 individuals, including 63 ALS patients and 51 healthy age- and sex-matched controls (Table 1). The ALS sample was composed by 27 patients with a family history of ALS, including 14 patients with mutation in the *SOD1* gene (detailed in Supplementary Table 1) and 13 patients with a pathogenic *C9orf72* repeat expansion, and by 36 sporadic cases, with no affected family members and negative for mutations in the major ALS genes, namely *SOD1*, *C9orf72*, *FUS*, and *TARDBP* (Table 1). All the included ALS patients fulfilled the El Escorial criteria [67] for probable or definite ALS and had been diagnosed by expert neurologists at the NEMO (NeuroMuscular Omnicentre) clinical center in Milan, Italy, or at the IRCCS Mondino Foundation in Pavia, Italy. Genomic DNA extraction for genetic analyses was performed using standard automated procedures. All the patients were offered genetic testing and counseling. The search for *SOD1*, *TARDBP*, *FUS*, and *C9orf72* gene mutations was performed as described elsewhere [68–70]. As normal controls, we recruited healthy volunteer subjects matched for age, sex, and ethnic background, but with no relationship to the ALS patients used in our study. Family history of ALS was ascertained excluding all subjects with even one relative who developed ALS or any other neurodegenerative disorder. Informed and written consent was obtained from each subject before inclusion in the study, and the study was approved by the Ethics Committee of the Pisa University Hospital (Protocol number 14767/2018). The study was performed in accordance with the Declaration of Helsinki.

### D-loop methylation analysis

An aliquot of 1 ml of peripheral blood was collected from each subject in EDTA tubes and stored at − 20 °C until DNA extraction. Genomic DNA was extracted using the QIAmp DNA blood Mini Kit (Qiagen, Milan, Italy, Catalog N° 51106) following the manufacturer’s protocol. The extracted DNA was quantified using a Nano Drop ND 2000c spectrophotometer (NanoDrop Thermo scientific). Five hundred nanograms of DNA from each sample were treated with sodium bisulfite in order to convert all unmethylated cytosines into uracil. The bisulfite conversion was performed using the Bisulfite-Gold kit (Zymo research, USA, Catalog N° D5007), following the manufacturer’s instructions. Pyrosequencing analyses were performed at the University of Exeter Medical School. Bisulfite pyrosequencing was used to quantify DNA methylation across three individual CpG sites in the Heavy (H)-strand of the D-loop region, spanning from nucleotide 16,417 to nucleotide 73 within mtDNA (GenBank: J01415.2). A single amplicon (226 bp) was generated using primers designed by means of the PyroMark Assay Design software 2.0 (Qiagen, UK) (Table 2). All samples were randomized to reduce batch effects. A 30 μl PCR was carried out using 3 μl of × 10 buffer B1, 2 μl of MgCl_2_ (25 mM), 0.3 μl of dNTP mix (10 mM), 1.5 μl of forward and primer mix (10 μM), 0.3 μl of HOT FIRE Pol (5 U/μl), 50 ng of bisulfite converted DNA, and 20.9 μl of water. PCR amplifications were performed with the following protocol: 1 cycle of 95 °C for 15 min, 40 cycles of 95 °C for 45 s, annealing temperature (62 °C) for 45 s and 72 °C for 3 min, followed by a final extension at 72 °C for 10 min. PCR products were purified and sequenced by pyrosequencing using the sequencing primer. To monitor the intra- and inter-plate variation, universal genomic DNA (random genomic DNA) was added to each plate.

**Table 2:** Primer sequences, amplicon size, and number of CpGs analyzed by means of pyrosequencing

Region	Primer forward	Primer reverse	Sequencing primer	Amplicon size	Number of CpGs
D-loop	Bio-5′TAGGATGAGGTAGGAATTAAAGATAGATA-3′	5′ACATCTAATTCCTACTTCAAAATCAT-3′	5′CAAATCTATCACCCTATTAA-3′	226 bp	3

DNA methylation was quantified using the PyroMark Q48 system (Qiagen, UK) following the manufacturer’s standard instructions and the PyroMark Q48 Autoprep 4.2.1 software. The methylation level is expressed using percentage 5-methylcytosine (5-mC), and, given the high correlation for methylation levels among the three CpGs sites analyzed (*r* = 0.56, *p* < 0.0001 between CpG1 and CpG2; *r* = 0.78, *p* < 0.0001 between CpG1 and CpG3; *r* = 0.87, *p* < 0.0001 between CpG2 and CpG3), for data analysis purposes the mean methylation levels of the three CpG sites included in the amplicon was used.

### Assessment of mitochondrial DNA copy number

In order to evaluate mtDNA copy number, 10 ng of total cellular DNA was used as input for quantitative PCR (qPCR). Primers amplifying a nuclear DNA region (hemoglobin subunit β) and a mtDNA region (chrM:3,313-3,322) were taken from the literature [71]. qPCR reactions were performed with a C1000™ Thermal Cycler equipped with a CFX 96™ Real-Time System (Bio-Rad) with the following conditions: 15 min at 95 °C, followed by 40 cycles of 30 s at 95 °C, 45 s at 55 °C, and 45 s at 72 °C. qPCR was performed in a final volume of 10 μl, containing 5 μl of master mix (Qiagen, Catalog No. 59445), 10 pmol of each primer, and 1 μl (10 ng) of DNA template. Each reaction was performed in triplicate. Threshold cycle (Ct) values were obtained with the Bio-Rad CFX Manager Software (Bio-Rad). To determine the mtDNA content relative to nDNA, the following equations were used [72]:

1 $$ \Delta \mathrm{Ct}=\mathrm{nDNA}\ \mathrm{Ct}-\mathrm{mtDNA}\ \mathrm{Ct} $$

2 $$ \mathrm{Relative}\ \mathrm{mtDNA}\ \mathrm{content}=2\times {2}^{\Delta \mathrm{Ct}} $$

### Statistical analyses

D-loop methylation data and mtDNA copy number were tested for normality using the Kolmogorov-Smirnov test. Demographic data, such as age at sampling and sex, were compared between groups by means of Student’s *t* test and Fisher’s exact test, respectively. Analysis of covariance (ANCOVA), including age at sampling and sex as covariates, was used to investigate differences in D-loop methylation levels among groups, followed by post hoc Bonferroni’s correction for multiple comparisons. Data are presented as mean ± SD. Pearson correlation coefficients were used to evaluate correlations between D-loop methylation levels, mtDNA copy number, and age at sampling. Statistical analyses were performed with STATGRAPHICS 5.1 plus software package for Windows and the MedCalc statistical software v. 12.5. Figures were obtained with GraphPad PRISM version 6.01.

## Supplementary information

**Additional file 1: Table S1.** Details of *SOD1* pathogenic variants (NM_000454) in ALS patients.

## Data Availability

The datasets generated and/or analyzed during the current study are available from the corresponding author on reasonable request.

## Abbreviations

5-hmC: 5-hydroxymethylcytosine
5-mC: 5-methylcytosine
AD: Alzheimer’s disease
ALS: Amyotrophic lateral sclerosis
*C9orf72*: Chromosome 9 open reading frame 72
CpG: Cytosine-phosphate-guanine site
D-loop: Displacement loop
DNMT1: DNA methyltransferase 1
*Dnmt3a*: DNA methyltransferase 3a
*FUS*: Fused in sarcoma
miRNA: MicroRNA
mtDNA: Mitochondrial DNA
*MT-RNR1*: Mitochondrially encoded 12S rRNA
nDNA: Nuclear DNA
OXPHOS: Oxidative phosphorylation
PD: Parkinson’s disease
q-PCR: Quantitative polymerase chain reaction
*SOD1*: Superoxide dismutase 1
*TARDBP*: TAR DNA-binding protein
TDP43: TAR DNA-binding protein 43

## References

[1] Smith EF, Shaw PJ, De Vos KJ (2019). The role of mitochondria in amyotrophic lateral sclerosis. Neurosci Lett.

[2] Brown RH, Al-Chalabi A (2017). Amyotrophic lateral sclerosis. N Engl J Med.

[3] Distad BJ, Weiss MD (2020). Edaravone for amyotrophic lateral sclerosis: more evidence for long-term benefit. Muscle Nerve.

[4] Bensimon G, Lacomblez L, Meininger V (1994). A controlled trial of riluzole in amyotrophic lateral sclerosis. ALS/Riluzole Study Group. N Engl J Med.

[5] WRITING GROUP ON BEHALF OF THE EDARAVONE (MCI-186) ALS 19 STUDY GROUP. Open-label 24-week extension study of edaravone (MCI-186) in amyotrophic lateral sclerosis. Amyotroph Lateral Scler Frontotemporal Degener. 2017. 10.1080/21678421.2017.1364269 28872920

[6] Chia R, Chiò A, Traynor BJ (2018). Novel genes associated with amyotrophic lateral sclerosis: diagnostic and clinical implications. Lancet Neurol.

[7] Yu B, Pamphlett R (2017). Environmental insults: critical triggers for amyotrophic lateral sclerosis. Transl Neurodegener.

[8] Nowicka N, Juranek J, Juranek JK, Wojtkiewicz J (2019). Risk factors and emerging therapies in amyotrophic lateral sclerosis. Int J Mol Sci.

[9] Dolinar A, Ravnik-Glavač M, Glavač D (2018). Epigenetic mechanisms in amyotrophic lateral sclerosis: a short review. Mech Ageing Dev.

[10] Coppedè F, Stoccoro A, Mosca L, Gallo R, Tarlarini C, Lunetta C (2018). Increase in DNA methylation in patients with amyotrophic lateral sclerosis carriers of not fully penetrant SOD1 mutations. Amyotroph Lateral Scler Frontotemporal Degener.

[11] Figueroa-Romero C, Hur J, Bender DE, Delaney CE, Cataldo MD, Smith AL (2012). Identification of epigenetically altered genes in sporadic amyotrophic lateral sclerosis. PLoS One.

[12] Masala A, Sanna S, Esposito S, Rassu M, Galioto M, Zinellu A (2018). Epigenetic changes associated with the expression of amyotrophic lateral sclerosis (ALS) causing genes. Neuroscience..

[13] Morahan JM, Yu B, Trent RJ, Pamphlett R (2009). A genome-wide analysis of brain DNA methylation identifies new candidate genes for sporadic amyotrophic lateral sclerosis. Amyotroph Lateral Scler.

[14] Tarr IS, McCann EP, Benyamin B, Peters TJ, Twine NA, Zhang KY (2019). Monozygotic twins and triplets discordant for amyotrophic lateral sclerosis display differential methylation and gene expression. Sci Rep.

[15] Tremolizzo L, Messina P, Conti E, Sala G, Cecchi M, Airoldi L (2014). Whole-blood global DNA methylation is increased in amyotrophic lateral sclerosis independently of age of onset. Amyotroph Lateral Scler Frontotemporal Degener.

[16] Coppedè F, Stoccoro A (2019). Mitoepigenetics and neurodegenerative diseases. Front Endocrinol (Lausanne).

[17] Baccarelli AA, Byun HM. Platelet mitochondrial DNA methylation: a potential new marker of cardiovascular disease. Clin Epigenetics. 2015:7–44. 10.1186/s13148-015-0078-0 PMC4404685 25901189

[18] Feng S, Xiong L, Ji Z, Cheng W, Yang H (2012). Correlation between increased ND2 expression and demethylated displacement loop of mtDNA in colorectal cancer. Mol Med Rep.

[19] Blanch M, Mosquera JL, Ansoleaga B, Ferrer I, Barrachina M (2016). Altered mitochondrial DNA methylation pattern in Alzheimer disease-related pathology and in Parkinson disease. Am J Pathol.

[20] Stoccoro A, Siciliano G, Migliore L, Coppedè F (2017). Decreased methylation of the mitochondrial D-loop region in late-onset Alzheimer’s disease. J Alzheimers Dis.

[21] Xu Y, Xu L, Han M, Liu X, Li F, Zhou X (2019). Altered mitochondrial DNA methylation and mitochondrial DNA copy number in an APP/PS1 transgenic mouse model of Alzheimer disease. Biochem Biophys Res Commun.

[22] Wong M, Gertz B, Chestnut BA, Martin LJ (2013). Mitochondrial DNMT3A and DNA methylation in skeletal muscle and CNS of transgenic mouse models of ALS. Front Cell Neurosci.

[23] Stoccoro A, Mosca L, Carnicelli V, Cavallari U, Lunetta C, Marocchi A (2018). Mitochondrial DNA copy number and D-loop region methylation in carriers of amyotrophic lateral sclerosis gene mutations. Epigenomics..

[24] Onesto E, Colombrita C, Gumina V, Borghi MO, Dusi S, Doretti A (2016). Gene-specific mitochondria dysfunctions in human TARDBP and C9ORF72 fibroblasts. Acta Neuropathol Commun.

[25] Pansarasa O, Bordoni M, Drufuca L, Diamanti L, Sproviero D, Trotti R (2018). Lymphoblastoid cell lines as a model to understand amyotrophic lateral sclerosis disease mechanisms. Dis Model Mech.

[26] Ebbert MTW, Ross CA, Pregent LJ, Lank RJ, Zhang C, Katzman RB (2017). Conserved DNA methylation combined with differential frontal cortex and cerebellar expression distinguishes C9orf72-associated and sporadic ALS, and implicates SERPINA1 in disease. Acta Neuropathol.

[27] Tibshirani M, Tradewell ML, Mattina KR, Minotti S, Yang W, Zhou H (2015). Cytoplasmic sequestration of FUS/TLS associated with ALS alters histone marks through loss of nuclear protein arginine methyltransferase 1. Hum Mol Genet.

[28] Rouaux C, Jokic N, Mbebi C, Boutillier S, Loeffler JP, Boutillier AL (2003). Critical loss of CBP/p300 histone acetylase activity by caspase-6 during neurodegeneration. EMBO J.

[29] Jun MH, Ryu HH, Jun YW, Liu T, Li Y, Lim CS (2017). Sequestration of PRMT1 and Nd1-L mRNA into ALS-linked FUS mutant R521C-positive aggregates contributes to neurite degeneration upon oxidative stress. Sci Rep.

[30] Liu Y, Wang J (2019). C9orf72-dependent lysosomal functions regulate epigenetic control of autophagy and lipid metabolism. Autophagy..

[31] Prasad A, Bharathi V, Sivalingam V, Girdhar A, Patel BK (2019). Molecular mechanisms of TDP-43 misfolding and pathology in amyotrophic lateral sclerosis. Front Mol Neurosci.

[32] Bianchessi V, Vinci MC, Nigro P, Rizzi V, Farina F, Capogrossi MC (2016). Methylation profiling by bisulfite sequencing analysis of the mtDNA non-coding region in replicative and senescent endothelial cells. Mitochondrion..

[33] van der Wijst MG, van Tilburg AY, Ruiters MH, Rots MG (2017). Experimental mitochondria-targeted DNA methylation identifies GpC methylation, not CpG methylation, as potential regulator of mitochondrial gene expression. Sci Rep.

[34] Byun HM, Panni T, Motta V, Hou L, Nordio F, Apostoli P (2013). Effects of airborne pollutants on mitochondrial DNA methylation. Part Fibre Toxicol.

[35] Sanyal T, Bhattacharjee P, Bhattacharjee S, Bhattacharjee P (2018). Hypomethylation of mitochondrial D-loop and ND6 with increased mitochondrial DNA copy number in the arsenic-exposed population. Toxicology..

[36] Xu Y, Li H, Hedmer M, Hossain MB, Tinnerberg H, Broberg K (2017). Occupational exposure to particles and mitochondrial DNA—relevance for blood pressure. Environ Health.

[37] Zheng LD, Linarelli LE, Liu L, Wall SS, Greenawald MH, Seidel RW (2015). Insulin resistance is associated with epigenetic and genetic regulation of mitochondrial DNA in obese humans. Clin Epigenetics.

[38] Gao J, Wen S, Zhou H, Feng S (2015). De-methylation of displacement loop of mitochondrial DNA is associated with increased mitochondrial copy number and nicotinamide adenine dinucleotide subunit 2 expression in colorectal cancer. Mol Med Rep.

[39] Janssen BG, Byun HM, Gyselaers W, Lefebvre W, Baccarelli AA, Nawrot TS (2015). Placental mitochondrial methylation and exposure to airborne particulate matter in the early life environment: an ENVIRONAGE birth cohort study. Epigemnetics..

[40] Fernández-Silva P, Enriquez JA, Montoya J (2003). Replication and transcription of mammalian mitochondrial DNA. Exp Physiol.

[41] Mposhi A, Van der Wijst MG, Faber KN, Rots MG (2017). Regulation of mitochondrial gene expression, the epigenetic enigma. Front Biosci (Landmark Ed).

[42] Shock LS, Thakkar PV, Peterson EJ, Moran RG, Taylor SM (2011). DNA methyltransferase 1, cytosine methylation, and cytosine hydroxymethylation in mammalian mitochondria. Proc Natl Acad Sci U S A.

[43] Mishra M, Kowluru RA (2015). Epigenetic modification of mitochondrial DNA in the development of diabetic retinopathy. Invest Ophthalmol Vis Sci.

[44] Liu YF, Zhu JJ, Yu Tian X, Liu H, Zhang T, Zhang YP (2020). Hypermethylation of mitochondrial DNA in vascular smooth muscle cells impairs cell contractility. Cell Death Dis.

[45] Tong H, Zhang L, Gao J, Wen S, Zhou H, Feng S (2017). Methylation of mitochondrial DNA displacement loop region regulates mitochondrial copy number in colorectal cancer. Mol Med Rep.

[46] Novielli C, Mandò C, Tabano S, Anelli GM, Fontana L, Antonazzo P (2017). Mitochondrial DNA content and methylation in fetal cord blood of pregnancies with placental insufficiency. Placenta..

[47] Kim BW, Jeong YE, Wong M, Martin LJ (2020). DNA damage accumulates and responses are engaged in human ALS brain and spinal motor neurons and DNA repair is activatable in iPSC-derived motor neurons with SOD1 mutations. Acta Neuropathol Commun.

[48] Shrivastava M, Vivekanandhan S, Pati U, Behari M, Das TK (2011). Mitochondrial perturbance and execution of apoptosis in platelet mitochondria of patients with amyotrophic lateral sclerosis. Int J Neurosci.

[49] Ladd AC, Keeney PM, Govind MM, Bennett JP (2014). Mitochondrial oxidative phosphorylation transcriptome alterations in human amyotrophic lateral sclerosis spinal cord and blood. NeuroMolecular Med.

[50] Ehinger JK, Morota S, Hansson MJ, Paul G, Elmér E (2015). Mitochondrial dysfunction in blood cells from amyotrophic lateral sclerosis patients. J Neurol.

[51] Masser DR, Clark NW, Van Remmen H, Freeman WM (2016). Loss of the antioxidant enzyme CuZnSOD (Sod1) mimics an age-related increase in absolute mitochondrial DNA copy number in the skeletal muscle. Age (Dordr).

[52] Dzitoyeva S, Chen H, Manev H (2012). Effect of aging on 5-hydroxymethylcytosine in brain mitochondria. Neurobiol Aging.

[53] D'Aquila P, Giordano M, Montesanto A, De Rango F, Passarino G, Bellizzi D (2015). Age- and gender-related pattern of methylation in the MT-RNR1 gene. Epigenomics..

[54] Mawlood SK, Dennany L, Watson N, Dempster J, Pickard BS (2016). Quantification of global mitochondrial DNA methylation levels and inverse correlation with age at two CpG sites. Aging (Albany NY).

[55] Ratnu VS, Emami MR, Bredy TW (2017). Genetic and epigenetic factors underlying sex differences in the regulation of gene expression in the brain. J Neurosci Res.

[56] Klinge CM (2020). Estrogenic control of mitochondrial function. Redox Biol.

[57] Mengel-From J, Svane AM, Pertoldi C, Nygaard Kristensen T, Loeschcke V, Skytthe A (2019). Advanced parental age at conception and sex affects mitochondrial DNA copy number in human and fruit flies. J Gerontol A Biol Sci Med Sci.

[58] Skuratovskaia D, Litvinova L, Vulf M, Zatolokin P, Popadin K, Mazunin I (2019). From normal to obesity and back: the associations between mitochondrial DNA copy number, gender, and body mass index. Cells..

[59] Wu J, Basha MR, Brock B, Cox DP, Cardozo-Pelaez F, McPherson CA (2008). Alzheimer’s disease (AD)-like pathology in aged monkeys after infantile exposure to environmental metal lead (Pb): evidence for a developmental origin and environmental link for AD. J Neurosci.

[60] Eid A, Bihaqi SW, Renehan WE, Zawia NH (2016). Developmental lead exposure and lifespan alterations in epigenetic regulators and their correspondence to biomarkers of Alzheimer’s disease. Alzheimers Dement (Amst).

[61] Manczak M, Park BS, Jung Y, Reddy PH (2004). Differential expression of oxidative phosphorylation genes in patients with Alzheimer’s disease: implications for early mitochondrial dysfunction and oxidative damage. NeuroMolecular Med.

[62] Liang WS, Reiman EM, Valla J, Dunckley T, Beach TG, Grover A (2008). Alzheimer’s disease is associated with reduced expression of energy metabolism genes in posterior cingulate neurons. Proc Natl Acad Sci U S A.

[63] Lunnon K, Ibrahim Z, Proitsi P, Lourdusamy A, Newhouse S, Sattlecker M (2012). Mitochondrial dysfunction and immune activation are detectable in early Alzheimer’s disease blood. J Alzheimers Dis.

[64] Lunnon K, Keohane A, Pidsley R, Newhouse S, Riddoch-Contreras J, Thubron EB (2017). Mitochondrial genes are altered in blood early in Alzheimer’s disease. Neurobiol Aging.

[65] Dal Canto MC, Gurney ME (1994). Development of central nervous system pathology in a murine transgenic model of human amyotrophic lateral sclerosis. Am J Pathol.

[66] Vande Velde C, McDonald KK, Boukhedimi Y, McAlonis-Downes M, Lobsiger CS, Bel Hadj S (2011). Misfolded SOD1 associated with motor neuron mitochondria alters mitochondrial shape and distribution prior to clinical onset. PLoS One.

[67] Brooks BR, Miller RG, Swash M, Munsat TL, World Federation of Neurology Research Group on Motor Neuron Diseases (2000). El Escorial revisited: revised criteria for the diagnosis of amyotrophic lateral sclerosis. Amyotroph Lateral Scler Other Motor Neuron Disord.

[68] Millecamps S, Salachas F, Cazeneuve C, Gordon P, Bricka B, Camuzat A (2010). SOD1, ANG, VAPB, TARDBP, and FUS mutations in familial amyotrophic lateral sclerosis: genotype-phenotype correlations. J Med Genet.

[69] Renton AE, Majounie E, Waite A, Simón-Sánchez J, Rollinson S, Gibbs JR (2011). A hexanucleotide repeat expansion in C9ORF72 is the cause of chromosome 9p21-linked ALS-FTD. Neuron..

[70] Tarlarini C, Lunetta C, Mosca L, Avemaria F, Riva N, Mantero V (2015). Novel FUS mutations identified through molecular screening in a large cohort of familial and sporadic amyotrophic lateral sclerosis. Eur J Neurol.

[71] Byun HM, Barrow TM (2015). Analysis of pollutant-induced changes in mitochondrial DNA methylation. Methods Mol Biol.

[72] Rooney JP, Ryde IT, Sanders LH, Howlett EH, Colton MD, Germ KE (2015). PCR based determination of mitochondrial DNA copy number in multiple species. Methods Mol Biol.

