# Supplementary material for: Reduced mitochondrial D-loop methylation levels in sporadic amyotrophic lateral sclerosis
Source: Clin Epigenetics. 2020 Sep 11;12:137. doi: 10.1186/s13148-020-00933-2 (PMC7488473; doi:10.1186/s13148-020-00933-2)
Supplement: Supplementary file 1 — Additional file 1: Table S1. Details of SOD1 pathogenic variants (NM_000454) in ALS patients. [file 13148_2020_933_MOESM1_ESM.docx]

| Nucleotide change | Amino acid change | Number of subjects (total n = 14) |
| --- | --- | --- |
| c.16G>A | **p.Val6Met** | 1 |
| c.197A>G | **p.Asn66Ser** | 1 |
| c.255G>T | **p.Leu85Phe** | 1 |
| c.272A>C | **p.Asp91Ala** | 3 |
| c.281G>A | **p.Gly94Asp** | 1 |
| c.365A>G | **p.Glu122Gly** | 1 |
| c.400_402delGAA | **p.Glu134del** | 1 |
| c.412A>G | **p.Thr138Ala** | 1 |
| c.435G>C | **p.Leu145Phe** | 2 |
| c.449T>C | **p.Ile150Thr** | 1 |
| c.461A>G | **p.Gln154Arg** | 1 |
